# Brainstem Hypoxia Contributes to the Development of Hypertension in the Spontaneously Hypertensive Rat

**DOI:** 10.1161/HYPERTENSIONAHA.114.04683

**Published:** 2015-03-11

**Authors:** Nephtali Marina, Richard Ang, Asif Machhada, Vitaliy Kasymov, Anastassios Karagiannis, Patrick S. Hosford, Valentina Mosienko, Anja G. Teschemacher, Pirkko Vihko, Julian F. R. Paton, Sergey Kasparov, Alexander V. Gourine

**Affiliations:** From the Centre for Cardiovascular and Metabolic Neuroscience (N.M., R.A., A.M., V.K., A.K., P.S.H., A.V.G.), Department of Clinical Pharmacology and Experimental Therapeutics (N.M., P.S.H.), and Neuroscience, Physiology and Pharmacology (R.A., A.M., V.K., A.K., A.V.G.), University College London, London, United Kingdom; School of Physiology and Pharmacology, University of Bristol, Bristol, United Kingdom (V.M., A.G.T., J.F.R.P., S.K.); and Department of Clinical Chemistry, University of Helsinki, Helsinki, Finland (P.V.).

**Keywords:** adenosine triphosphate, hypertension, hypoxia, lactic acid, sympathetic nervous system

## Abstract

Systemic arterial hypertension has been previously suggested to develop as a compensatory condition when central nervous perfusion/oxygenation is compromised. Principal sympathoexcitatory C1 neurons of the rostral ventrolateral medulla oblongata (whose activation increases sympathetic drive and the arterial blood pressure) are highly sensitive to hypoxia, but the mechanisms of this O_2_ sensitivity remain unknown. Here, we investigated potential mechanisms linking brainstem hypoxia and high systemic arterial blood pressure in the spontaneously hypertensive rat. Brainstem parenchymal PO_2_ in the spontaneously hypertensive rat was found to be ≈15 mm Hg lower than in the normotensive Wistar rat at the same level of arterial oxygenation and systemic arterial blood pressure. Hypoxia-induced activation of rostral ventrolateral medulla oblongata neurons was suppressed in the presence of either an ATP receptor antagonist MRS2179 or a glycogenolysis inhibitor 1,4-dideoxy-1,4-imino-d-arabinitol, suggesting that sensitivity of these neurons to low PO2 is mediated by actions of extracellular ATP and lactate. Brainstem hypoxia triggers release of lactate and ATP which produce excitation of C1 neurons in vitro and increases sympathetic nerve activity and arterial blood pressure in vivo. Facilitated breakdown of extracellular ATP in the rostral ventrolateral medulla oblongata by virally-driven overexpression of a potent ectonucleotidase transmembrane prostatic acid phosphatase results in a significant reduction in the arterial blood pressure in the spontaneously hypertensive rats (but not in normotensive animals). These results suggest that in the spontaneously hypertensive rat, lower PO_2_ of brainstem parenchyma may be associated with higher levels of ambient ATP and l-lactate within the presympathetic circuits, leading to increased central sympathetic drive and concomitant sustained increases in systemic arterial blood pressure.

## Abstract 2

Supplemental Digital Content is available in the text.

Hypertension is one of the main risk factors for the development of many cardiovascular diseases. Despite significant progress in the diagnosis and treatment of hypertension, approximately only half of patients show satisfactory response to treatment.^1^ This poor efficacy might be because of the fact that conventional antihypertensive therapies are aimed at downstream peripheral mechanisms which maintain high systemic arterial blood pressure, while primary factors responsible for the development of the condition remain untreated.

The pathophysiology of systemic arterial hypertension is complex and in general poorly understood, but over the last 3 decades studies in animal models and patients with hypertension have provided significant evidence that activation of the sympathetic nervous system is linked to the development and maintenance of the condition.^2–4^ Vasomotor and cardiac activities of spinal sympathetic preganglionic neurons depend on tonic descending excitatory drive generated by sympathoexcitatory (presympathetic) neuronal networks residing in the hypothalamus and the brainstem^5–9^: the rostral ventrolateral medulla (RVLM), rostral ventromedial and midline medulla, the A5 cell group of the pons, and the paraventricular hypothalamic nucleus.^6,8,10–12^ Bulbospinal neurons of the RVLM which belong to the catecholaminergic C1 group are believed to be of a prime importance for the maintenance of vasomotor sympathetic tone.^12^

One of the potential mechanisms which may be responsible for sympathetic activation in hypertension is based on the operation of a so-called Cushing response characterized by a triad of high blood pressure, irregular breathing, and bradycardia.^13^ Although originally described as an autoresuscitation mechanism recruited under extreme pathological conditions such as brain ischemia, it is currently viewed as a physiological compensatory response to compromised brain perfusion or brain hypoxia.^14,15^ Brainstem vasculature of patients with hypertension and of animal models of hypertension (eg, spontaneously hypertensive rat [SHR]) is considerably narrower (compared with the respective normotensive counterparts), resulting in high cerebral artery resistance.^16,17^ This is not a consequence of hypertension as it occurs prior to its development, at least in the SHR.^16^ Thus, the neuronal sympathoexcitatory networks that control the arterial blood pressure have been suggested to be hypoperfused/hypoxic.^16,18^ By increasing systemic arterial blood pressure in response to compromised brainstem perfusion, the Cushing mechanism would be expected to produce changes in the circulatory system to preserve oxygen delivery and maintain brain oxygenation at the expense of systemic hypertension.^15,16,18,19^

The mechanisms underlying sympathetic activation associated with compromised brain tissue perfusion and hypoxia remain unknown. Presympathetic RVLM neurons are highly sensitive to hypoxia,^20–22^ but the mechanisms of their O_2_ sensitivity have never been addressed. Brain tissue hypoxia is expected to be associated with increases in the level of extracellular lactate, which was recently shown to have a profound excitatory effect on another notable population of brainstem catecholaminergic neurons residing in the pontine locus coeruleus.^23^ In addition, we have previously shown that hypoxia triggers release of ATP within the RVLM.^24^ Other pharmacological studies have revealed that activation of ATP receptors in the RVLM by microinjections of ATP or stable ATP analogues increases the excitability of C1 neurons and leads to the increases in the arterial blood pressure, heart rate, and renal sympathetic nerve activity.^25–27^

This study was designed to test the hypothesis that brainstem hypoxia is associated with the pathogenesis of systemic arterial hypertension. We measured oxygen tension from within the presympathetic RVLM region of the brainstem in the SHRs and control Wistar rats, determined whether oxygen sensitivity of presympathetic C1 neurons is direct or mediated by prior release and actions of ATP and lactate, evaluated the effects of l-lactate on sympathetic nerve activity and the arterial blood pressure, and determined the effect of blocking ATP-mediated signaling in the RVLM on systemic arterial blood pressure in the SHRs and their normotensive counterparts.

## Methods

All animal experimentations were performed in accordance with the European Commission Directive 86/609/EEC (European Convention for the Protection of Vertebrate Animals used for Experimental and Other Scientific Purposes) and the UK Home Office (Scientific Procedures) Act (1986) with project approval from the respective Institutional Animal Care and Use Committees. Detailed description of the Materials and Methods used in the current study is available in the online-only Data Supplement.

## Results

### Brainstem of the SHR Is Hypoxic at Normal Levels of the Arterial Blood Pressure

**Figure 1.**
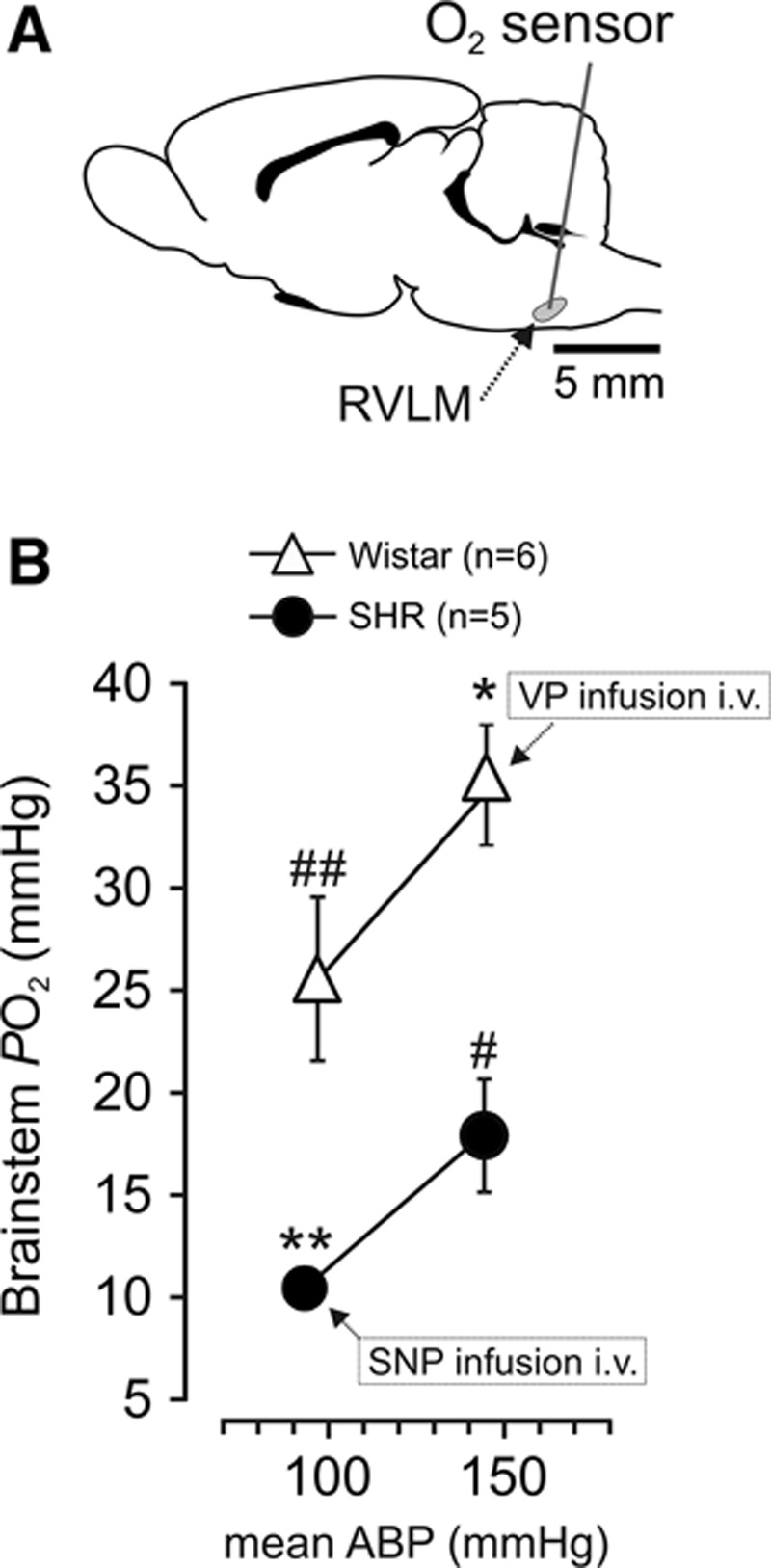
Brainstem of the spontaneously hypertensive rat (SHR) is hypoxic at physiological levels of the systemic arterial blood pressure. **A**, Schematic drawing of the rat brain in sagittal projection illustrating the site of PO_2_ measurements taken from the anatomic location of the presympathetic circuits of the rostral ventrolateral medulla (RVLM). **B**, Summary data showing parenchymal PO_2_ levels in the RVLM of anesthetized SHRs and Wistar rats. PO_2_ measurements were taken before and during intravenous infusion of sodium nitroprusside (SNP) and vasopressin (VP) in SHRs and Wistar rats, respectively. Data are presented as means±SEM. Note that because the arterial blood pressure in these experiments was clamped, SE values are small and the respective error bars are embedded within the symbols. Paired *t* test: RVLM PO_2_ in Wistar rats, resting vs vasopressin (*P=0.019); RVLM PO_2_ in SHRs, resting vs SNP (**P=0.006). Unpaired *t* test: RVLM PO_2_ in SHRs vs hypertensive Wistar rats (#*P*=0.006); RVLM PO_2_ in normotensive Wistar rats vs normotensive SHRs (##*P*=0.025).

At baseline conditions, ventrolateral medullary PO_2_ in anesthetized SHRs was found to be slightly lower compared with their age- and sex-matched normotensive Wistar rats (18±3 mm Hg [n=5] versus 26±4 mm Hg [n=6]; *P*=0.07; Figure 1B). When the arterial blood pressure of anesthetized SHRs was lowered to the level of normotensive Wistar rats (from 144±3 mm Hg to 93±1 mm Hg) by intravenous infusion of sodium nitroprusside (arterial PO_2_ level was kept at ≈100 mm Hg), brainstem PO_2_ decreased to 11±1 mm Hg (*P*=0.006, paired *t* test, RVLM PO_2_ in the SHRs at baseline versus in normotensive conditions; Figure 1B). When mean arterial blood pressure of Wistar rats was increased to the level of SHRs (from 97±1 mm Hg to 145±5 mm Hg) by intravenous infusion of vasopressin (while keeping arterial PO_2_ level at ≈100 mm Hg), brainstem PO_2_ increased to 35±3 mm Hg (*P*=0.019, paired *t* test, RVLM PO_2_ in Wistar rats at baseline versus in hypertensive conditions; Figure 1B). Thus, resting RVLM PO_2_ in SHRs is lower than that of hypertensive Wistar rats (*P*=0.006, unpaired *t* test; Figure 1B), and resting RVLM PO_2_ in Wistar rats is higher than that of normotensive SHRs (*P*=0.025, unpaired *t* test; Figure 1B). Similar data were obtained when tissue PO_2_ measurements were taken from within the dorsal medullary nucleus of the solitary tract. Baseline PO_2_ in the nucleus of the solitary tract of anesthetized SHRs (n=3) was 16±2 mm Hg decreasing to 8±3 mm Hg (*P*<0.05, paired *t* test) when the arterial blood pressure was lowered to the normotensive level. In age- and sex-matched normotensive Wistar rats (n=3), baseline nucleus of the solitary tract PO_2_ was 19±3 mm Hg (n=3) increasing to 30±5 mm Hg (*P*<0.05, paired *t* test) when blood pressure was increased to the level recorded in the SHRs. These data confirm that the level of the arterial blood pressure determines PO_2_ of the brainstem parenchyma. The data also demonstrate that the brainstem PO_2_ in the SHR is ≈15 mm Hg lower than in the Wistar rat at the same level of the systemic arterial blood pressure.

### ATP and Lactate Mediate Excitation of RVLM Neurons at Low PO_2_

**Figure 2.**
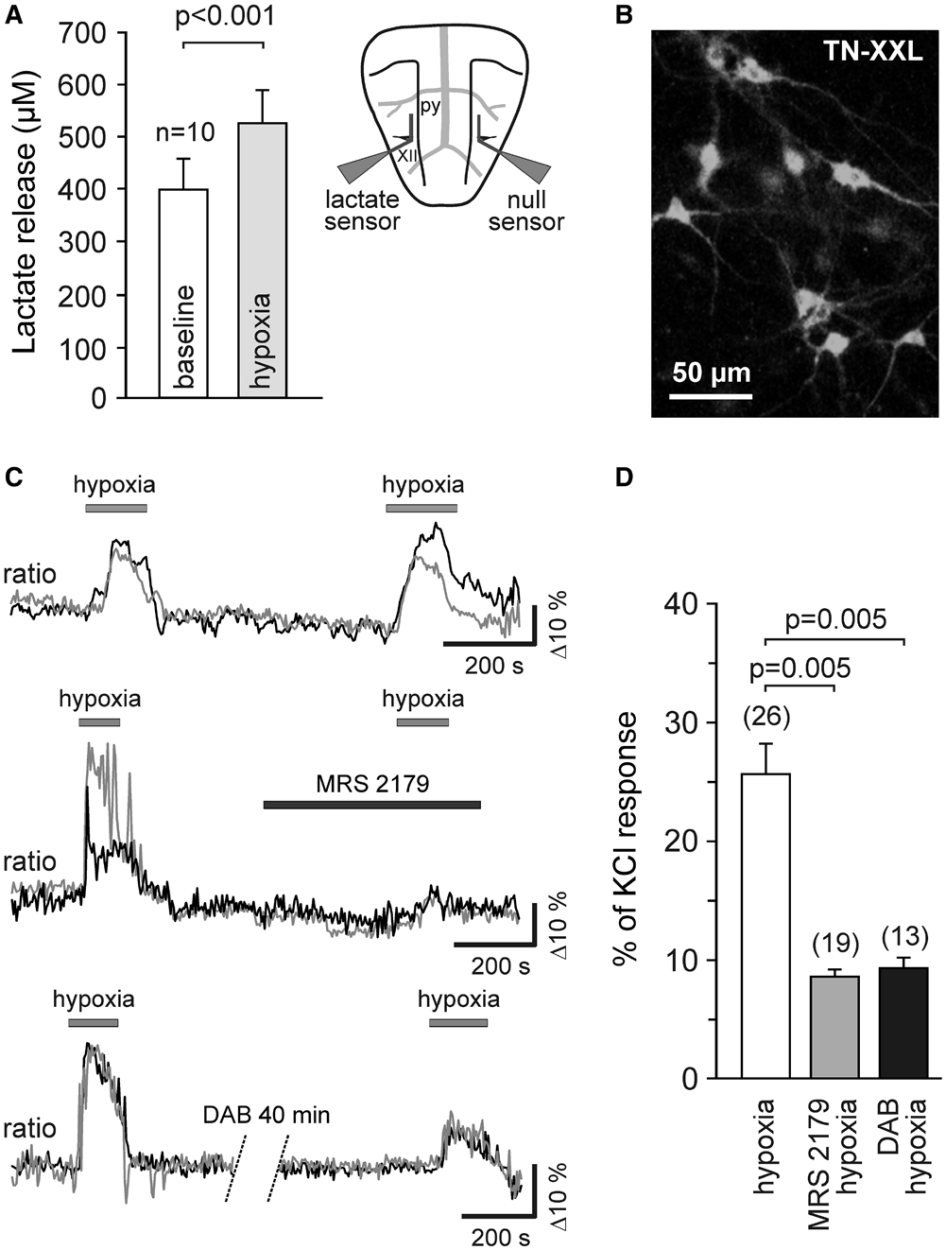
Release and actions of ATP and lactate mediate activation of rostral ventrolateral medulla (RVLM) neurons during hypoxia. **A**, Summary data obtained in vitro using horizontal slices of the rat brainstem showing tonic release of lactate from the ventral surface of the medulla oblongata and peak lactate release during hypoxia. Inset, Schematic drawing of a horizontal brainstem slice illustrating dual recording configuration of lactate and null (control) biosensors placed on the ventral medullary surface. Difference in current between lactate and null biosensors was used to determine the amount of lactate release. py, pyramidal tract; XII hypoglossal rootlets. **B**, Putative presympathetic C1 RVLM neurons visualized in organotypic brainstem slices after transduction with an adenoviral vector to express genetically encoded Ca^2+^ indicator TN-XXL under the control of PRSx8 promoter. **C**, Raw traces (changes in intracellular [Ca^2+^] of 2 individual neurons are shown on each plot) illustrating robust and reproducible responses of the RVLM neurons to hypoxia (**upper plot**) as well as the effects of ATP receptor antagonist MRS2179 (30 µmol/L) (**middle**) and glycogenolysis inhibitor 1,4-dideoxy-1,4-imino-d-arabinitol (DAB) (500 µmol/L) (**bottom**) on hypoxia-induced [Ca^2+^]_i_ responses of these neurons (ratiometric imaging using TN-XXL). **D**, Summary data illustrating the effects of MRS2179 and DAB on hypoxia-induced [Ca^2+^]_i_ responses of putative C1 neurons. Data are presented as means±SEM.

We next determined whether the sensitivity of RVLM neurons to hypoxia is direct or mediated by prior release and actions of ATP or lactate or both. In the acute brainstem slices of adult Wistar rats, hypoxia was associated with facilitated release of lactate (Figure 2A). Lactate biosensors placed in a direct contact with the ventral surface of the medulla oblongata detected tonic release of lactate of 396±58 µmol/L increasing to 507±71 µmol/L during 4 minutes of hypoxia (n=10; *P*<0.001, paired *t* test; Figure 2A). We demonstrated previously that release of ATP in the brainstem is also facilitated during hypoxia,^24^ and the next experiment examined whether the sensitivity of RVLM neurons to low PO_2_ is dependent on ATP- and lactate-mediated signaling.

Hypoxia-induced [Ca^2+^]_i_ responses in putative C1 neurons were visualized in organotypic brainstem slices using the genetically encoded Ca^2+^ indicator TN-XXL expressed under the control of the PRSx8 promoter (Figure 2B). Confirming previously reported data,^20,21^ hypoxia triggered robust and reproducible [Ca^2+^]_i_ elevations in 100% of the recorded neurons (Figure 2C). Hypoxia-induced [Ca^2+^]_i_ responses of these putative C1 neurons were markedly reduced in the presence of an ATP receptor antagonist MRS2179 (30 µmol/L; n=19, *P*=0.005, paired *t* test; Figure 2C and 2D) or after incubation of the slice with glycogenolysis inhibitor 1,4-dideoxy-1,4-imino-d-arabinitol (500 µmol/L; n=13, *P*=0.005, paired *t* test; Figure 2C and 2D). These data suggest that oxygen sensitivity of RVLM neurons is not intrinsic but indirect, and mediated by actions of ATP and lactate released from as yet unknown cellular source, but most likely astrocytes.^23,28,29^

### l-Lactate Activates RVLM Neurons In Vitro and Induces Increases in the Sympathetic Nerve Activity and the Arterial Blood Pressure In Vivo

**Figure 3.**
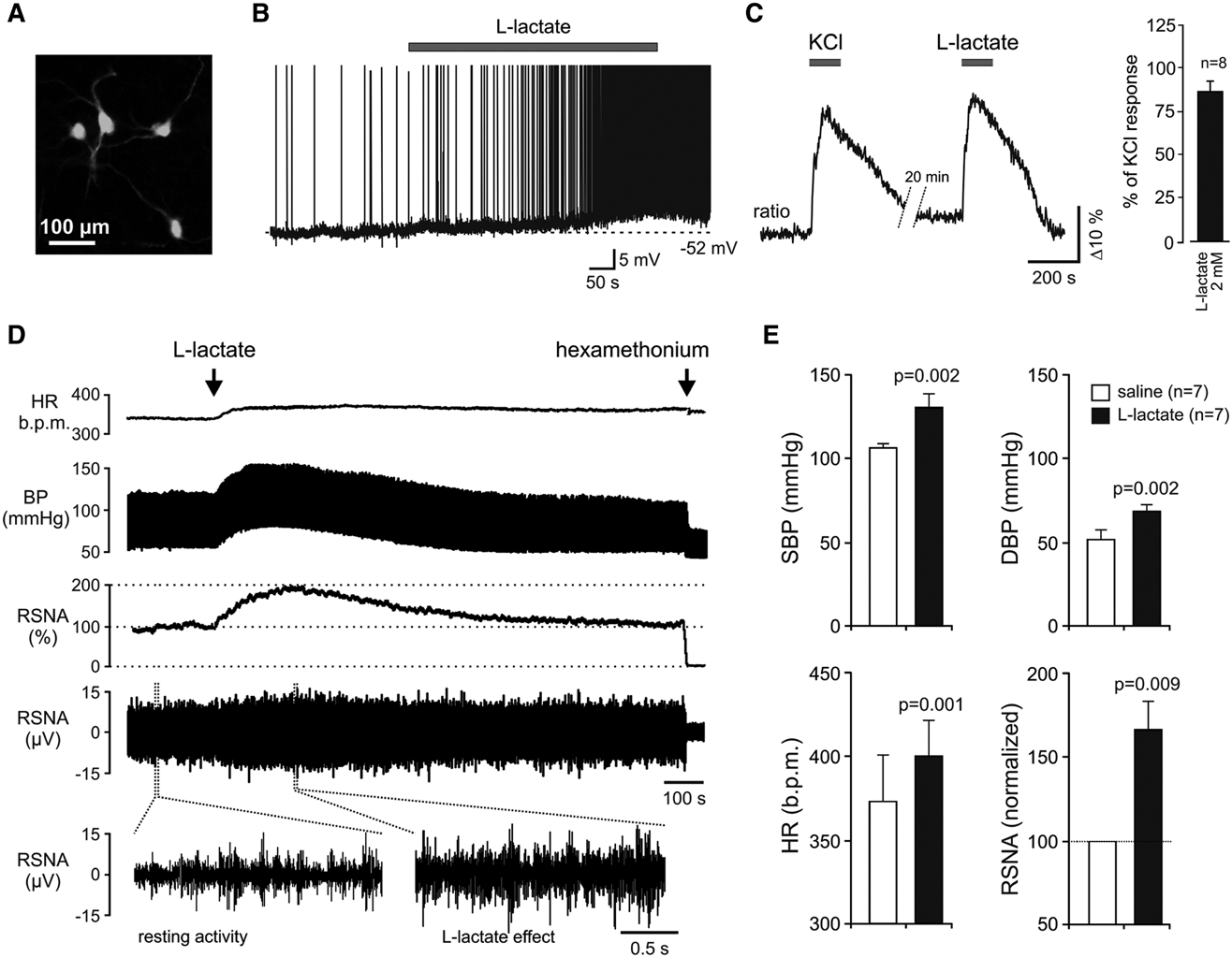
l-Lactate activates rostral ventrolateral medulla (RVLM) neurons in vitro and induces increases in the sympathetic nerve activity and the arterial blood pressure in vivo. **A**, Putative C1 neurons in organotypic brainstem slice culture transduced to express enhanced green fluorescent protein under the control of PRSx8 promoter and visualized for patch clamp recordings. **B**, Representative trace of the membrane potential changes and electrical activity of a putative C1 neuron showing depolarization and increased rate of action potential firing in response to application of l-lactate (2 mmol/L, pH 7.4). **C**, Representative trace and summary data (**right**) illustrating [Ca^2+^]_i_ responses of putative C1 neurons to application of l-lactate (2 mmol/L, pH 7.4) and KCl (20 mmol/L) (ratiometric imaging using TN-XXL). **D**, Raw data obtained in anesthetized and artificially ventilated rat showing changes in heart rate, arterial blood pressure, and sympathetic nerve activity induced by l-lactate (20 mmol/L, pH 7.4) applied on the ventral surface of the medulla oblongata. **E**, Summary data illustrating the effect of l-lactate applied on the ventral surface of the medulla oblongata on systolic blood pressure (SBP), diastolic blood pressure (DBP), heart rate (HR) and renal sympathetic nerve activity (RSNA). Data are presented as means±SEM. BP indicates arterial blood pressure.

Putative C1 neurons recorded in organotypic brainstem slices (Figure 3A) responded to bath application of l-lactate (2 mmol/L, pH 7.4) with sustained depolarization (V_m_ increased from −62±5 mV to −56±5 mV, n=4, *P*=0.006, paired *t* test) and increased rate of action potential firing (Figure 3B). l-lactate (2 mmol/L) also triggered robust [Ca^2+^]_i_ elevations in ≈60% (8 of 12 neurons that responded to KCl, 20 mmol/L) of the recorded putative C1 neurons expressing TN-XXL (Figure 3C). Lactate-induced [Ca^2+^] elevations in RVLM neurons were remarkably strong (average peak of the response reaching 80% of that triggered by KCl) with a complete recovery within ≈5 minutes of lactate washout (Figure 3C). In pentobarbital-anesthetized and artificially ventilated normotensive Wistar rats (end-tidal CO_2_ was kept at ≈3.5±0.5% throughout the experiments), application of l-lactate (20 mmol/L, 30 µL) on the ventral surface of the medulla oblongata induced profound and sustained (lasting for at least 10 minutes) sympathoexcitatory response, characterized by significant increases in renal sympathetic nerve activity (by 66±18%, *P*=0.009, paired *t* test), systolic arterial blood pressure (from 106±2 to 131±8 mm Hg, *P*=0.002, paired *t* test), diastolic arterial blood pressure (from 52±5 to 68±4 mm Hg, *P*=0.002, paired *t* test), and heart rate (from 376±26 to 398±24 beats min^−1^, *P*=0.001, paired *t* test; Figure 3D and 3E). These data are consistent with recent observations showing that central catecholaminergic neurons are highly sensitive to lactate^23^ and suggest that higher levels of ambient lactate in the RVLM may contribute to the increased activity of presympathetic C1 bulbospinal vasomotor neurons and concomitant sympathetic activation and hypertension observed in the SHR.

### Facilitated Breakdown of Extracellular ATP in the RVLM Reduces the Degree of Hypertension in the SHR

**Figure 4.**
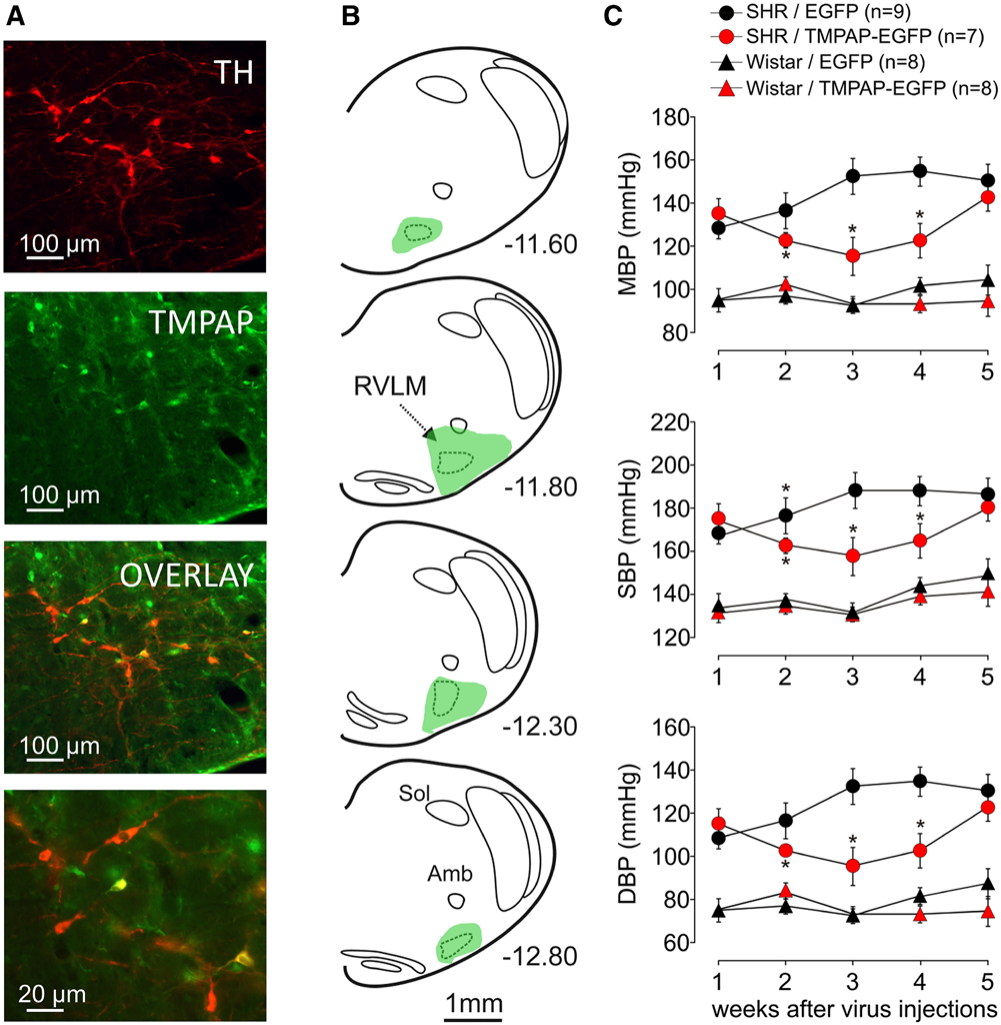
Transmembrane prostate acidic phosphatase (TMPAP) expression in the rostral ventrolateral medulla (RVLM) reduces the degree of hypertension in the spontaneously hypertensive rat (SHR). **A**, TMPAP–enhanced green fluorescent protein (EGFP) expression in the C1 region of the RVLM (TH, tyrosine hydroxylase). Bottom image is a high magnification micrograph showing a proportion of TH-positive neurons expressing TMPAP-EGFP. **B**, Schematic drawing of the rat brainstem in a series of coronal projections illustrating the representative extent of TMPAP expression in relation to the anatomic location of the RVLM presympathetic circuits. Numbers indicate distance from Bregma. TMPAP expression was highest in the ventrolateral medullary regions located at −11.80 mm from Bregma. **C**, Summary data showing that bilateral expression of TMPAP-EGFP within the C1 region of the RVLM results in a significant reduction of the arterial blood pressure in the SHR. The effect was sustained for 3 weeks. Note that TMPAP expression in the RVLM had no effect on the arterial blood pressure in Wistar rats. Data are presented as means±SEM. *Significant difference between SHRs expressing EGFP and SHRs expressing TMPAP-EGFP in the RVLM (*P*<0.05).

In the SHR, overexpression of a potent ectonucleotidase, transmembrane prostate acidic phosphatase (TMPAP), within the C1 area of the RVLM (Figure 4A and 4B; to promote facilitated ATP breakdown) was associated with a significant reduction in systemic arterial blood pressure (Figure 4C). This effect was maintained for 3 weeks of TMPAP expression in the RVLM [mean arterial blood pressure was 116±9 mm Hg (n=7) in the SHRs expressing TMPAP in the RVLM versus 153±8 mm Hg (n=9) in the SHRs expressing enhanced green fluorescent protein; *P*=0.009, Kruskal–Wallis ANOVA by ranks]. There were no differences in mean arterial blood pressure between 2 groups of SHRs 5 weeks after the injections of TMPAP or enhanced green fluorescent protein–expressing viral vectors (Figure 4C). TMPAP expression and activity in the RVLM had no effect on the arterial blood pressure of Wistar rats [TMPAP: 91±4 mm Hg (n=8) versus enhanced green fluorescent protein: 93±4 mm Hg (n=8); *P*=0.836, Kruskal–Wallis ANOVA by ranks]. Immunohistochemical analysis identified a proportion of tyrosine hydroxylase–positive (C1) RVLM neurons expressing TMPAP (Figure 4A) and a general strong expression of the transgene along the rostro-caudal extent of the RVLM (between −11.60 and −12.80 mm from Bregma; Figure 4B). Thus, TMPAP expression and facilitated breakdown of ATP in the RVLM lowers arterial blood pressure in the SHR without affecting this hemodynamic variable in control animals, suggesting that ATP-mediated signaling contribute to the maintenance of systemic arterial hypertension in this model.

## Discussion

This study tested the hypothesis that brainstem hypoxia contributes to the pathogenesis of systemic arterial hypertension. This hypothesis is difficult to test directly as this would require an experimental paradigm allowing differential precise manipulation of peripheral and cerebral oxygenation at different stages of development, including young prehypertensive SHRs. However, our evidence strongly supports the hypothesis. We found that the brainstem of the adult SHR is hypoxic (PO2≈10 mm Hg) at physiological levels of the arterial PO_2_ (100 mm Hg) and systemic arterial blood pressure (mean ≈95 mm Hg). PO_2_ measurements were taken from within the brainstem region that contains a major group of RVLM bulbospinal sympathoexcitatory (presympathetic) C1 neurons which generate vasomotor sympathetic tone.^7,12,30^ These neurons are known to be excited by low Po_2_,^20,21,31^ and our data indicate that their sensitivity to hypoxia is (at least partially) indirect (ie, nonintrinsic) and mediated by prior release and actions of ATP and lactate. Tissue hypoxia always leads to lactate release/accumulation^32^ (we confirm this in the current study, see Figure 2A), and here we demonstrate that lactate is capable of increasing the activity of RVLM neurons. We also found that application of lactate to the ventral surface of the brainstem in normotensive animals produces profound sympathoexcitation and increases in the arterial blood pressure. Hypoxia-evoked release of ATP and ATP stimulatory actions on C1 neurons, central sympathetic drive, and systemic arterial blood pressure have been demonstrated previously.^24,25,27^ Finally, in the SHR, blockade of ATP-mediated signaling in the RVLM presympathetic area by virally-driven overexpression of a potent ectonucleotidase (TMPAP), resulted in a significant lowering of the arterial blood pressure—the effect which was sustained for 3 weeks. The aim of this experiment was to achieve widespread and strong transgene expression in the general RVLM area. This would ensure effective ATP breakdown and blockade of ATP-mediated paracrine (volume) signaling. Although, only a proportion of the RVLM C1 neurons was transduced, expression of TMPAP by other cellular elements in the area was clearly sufficient to achieve significant reduction of the arterial blood pressure in the SHR. Together these data suggest that decreased parenchymal PO_2_ in the RVLM leads to higher levels of ambient ATP and lactate which increase the excitability of RVLM neurons, leading to enhanced vasomotor sympathetic tone and high systemic arterial blood pressure. We propose that in the SHR this serves as a compensatory condition needed to maintain adequate blood supply and oxygenation of the brain.

Several previous studies have shown that cerebral vascular resistance is high in patients with hypertension as well as in animal models of hypertension, including the SHRs.^14–17,33^ In rats, increasing cerebral vascular resistance was shown to raise sympathetic activity and the arterial blood pressure.^16^ Moreover, in the SHR, development of hypertension is associated with progressive impairment of neurovascular coupling, elevated baseline capillary red blood cell velocity,^34^ and impaired autoregulation because of vascular hypertrophy.^35^ Our data are consistent with these observations and directly demonstrate that the brainstem of SHRs becomes hypoxic when systemic arterial blood pressure decreases to physiological levels.

Histological analysis revealed robust TMPAP expression in the RVLM even at 6 weeks after the delivery of viral vectors (not shown). Therefore, fading TMPAP expression and declining activity are unlikely to be responsible for the recovery of the hypertensive phenotype in the SHR. Although TMPAP activity is effective in reducing systemic arterial blood pressure, our oxygen measurements suggest that this would also result in reduced cerebral oxygenation. We hypothesize that compensation is likely to develop over time to restore high arterial blood pressure by either recruitment of other mechanisms driving the activity of RVLM presympathetic neurons or by enhanced activity of presympathetic neurons residing in other regions of the central nervous system, not targeted to express TMPAP (eg, paraventricular nucleus). Moreover, at later stages of hypertension development in this model, other factors (renal and vascular) may become more significant in maintenance of the condition.

Earlier in vivo and in vitro studies have demonstrated that presympathetic C1 neurons are highly sensitive to hypoxia (or chemical hypoxia induced by CN^−^ application).^20,21,36–38^ Confirming these reports, hypoxia was found to trigger robust and reproducible [Ca^2+^]_i_ elevations in 100% of the recorded RVLM neurons. Hypoxia-induced responses in these putative C1 neurons were reduced to a similar extent either in the presence of an ATP receptor antagonist MRS2179 (which is acting preferentially at metabotropic P2Y_1_ receptors) or after incubation of the slice with glycogenolysis inhibitor 1,4-dideoxy-1,4-imino-d-arabinitol. These data suggest that sensitivity of C1 neurons to decreases in PO_2_ is mostly indirect and mediated by prior release and actions of ATP and lactate. In this study, we demonstrate directly that hypoxia leads to a significant increase in lactate efflux detected at the ventral surface of the brainstem, whereas our previous study reported profound hypoxia-induced release of ATP in the medulla oblongata—an effect observed in anesthetized and artificially ventilated rats and in acute brainstem slices in vitro.^24^

C1 neurons are known to be sensitive to ATP. ATP or stable ATP analogues increase their activity, triggers sympathoexcitation, and increases arterial blood pressure in normotensive rats.^25,27,39^ A role of purinergic signaling in modulating the activity of RVLM presympathetic circuits is also supported by a more recent evidence showing that P2Y_1_ receptor-mediated activation of C1 neurons contributes to sympathetic and blood pressure responses elicited by activation of the peripheral chemoreceptors.^40^ Furthermore, ATP-mediated signaling may also contribute to alterations in the central nervous mechanisms of autonomic control and development of hypertension in rats subjected to chronic intermittent hypoxia. This animal model shows significantly increased expression of ionotropic ATP receptors (P2X_3_ and P2X_4_ subunits in particular) in the RVLM^41^ and markedly enhanced sympathetic responses to microinjections of ATP into the RVLM.^42^

Responses of the RVLM neurons to lactate application were found to be similar in magnitude and time course to those observed recently in neurons of the pontine locus coeruleus, a major cluster of catecholaminergic cells in the central nervous system.^23^ Lactate is produced by activated astroglial cells and stimulates neurons of the locus coeruleus to release norepinephrine, and these effects are mimicked by application of exogenous lactate.^23^ As expected, hypoxia triggered release of lactate in the ventrolateral medulla (Figure 2A), and lactate produced potent sympathoexcitatory responses in vivo (Figure 3). We propose that interfering with lactate-mediated signaling could be a potential therapeutic strategy that may help to attenuate pathological sympathoexcitation associated with systemic arterial hypertension; however, because of current unavailability of a suitable blocker of lactate actions, we were not able to test this hypothesis in this study.

Nonexcitable cells in the brain (eg, astrocytes) communicate predominantly via release of ATP.^28,29,43^ Astrocytes are also the only type of brain cell which store glycogen^44^ and according to the lactate shuttle hypothesis^45^ supply energy to neurons in the form of lactate. Although the identity of cells which release ATP and lactate responsible for hypoxia-induced activation of RVLM neurons remains unknown, astroglia represents a plausible source. Astrocytes can detect various sensory modalities such as changes in brain parenchymal pH and glucose levels.^28,46^ C1 neurons are activated when RVLM astrocytes are selectively stimulated using an optogenetic approach.^43^ These astrocyte-driven responses of C1 neurons were found to be abolished in the presence of an ATP-degrading enzyme apyrase, suggesting that ATP mediates communication between astrocytes and these presympathetic neurons.^43^ Furthermore, in vivo experiments conducted in anesthetized and artificially ventilated rats showed that optogenetic stimulation of RVLM astrocytes increases sympathetic nerve activity and systemic arterial blood pressure.^43^

A major clinical problem associated with hypertension is stroke. Many strokes develop as a consequence of brain ischemia rather than hemorrhage. It is of interest that there is a significant association between hypertension before and after posterior cerebral artery infarction compared with anterior cerebral artery infarction.^17^ These observations in humans further support the hypothesis that brainstem hypoperfusion/hypoxia is an important contributor to the development of hypertension. Whether this is also the case in other cardiovascular diseases where central sympathetic drive is high is not known. However, we have recently shown that a similar mechanism involving facilitated ATP-mediated signaling in the RVLM contributes to sympathetic activation during the development of myocardial infarction-induced heart failure.^43^ Interestingly, a recent study using cerebral oximetry has demonstrated that cerebral tissue oxygen saturation in many heart failure patients is low, despite nearly normal levels measured in the arterial blood.^47^ It seems that the same mechanism activated by brainstem hypoxia might be responsible for heightened central sympathetic tone in both heart failure and systemic arterial hypertension.

### Perspectives

The data obtained in the present study suggest that in the SHR low parenchymal PO_2_ results in higher levels of ambient ATP and lactate within the presympathetic areas of the brainstem leading to the increased activity of sympathoexcitatory neurons and concomitant sustained increases in the arterial blood pressure.

## Sources of Funding

Experimental work described in this paper was funded by The Wellcome Trust (UK) and the British Heart Foundation. N. Marina is a British Heart Foundation Intermediate Basic Research Fellow (ref. FS/13/5/29927). A.V. Gourine is a Wellcome Trust Senior Research Fellow (ref. 095064).

## Disclosures

None.

## Novelty and Significance

### What Is New?

- Brainstem parenchymal oxygen tension in the spontaneously hypertensive rat is ≈15 mm Hg lower than in the normotensive Wistar rat at the same level of arterial oxygenation and systemic arterial blood pressure.
- Hypoxia-induced activation of presympathetic brainstem circuits is mediated by actions of ATP and lactate released in low PO_2_ conditions.
- Sustained blockade of ATP-mediated signaling within the presympathetic brainstem circuits results in a significant decrease in systemic arterial blood pressure in the spontaneously hypertensive rat (but not in normotensive animals).
- These findings are the first of its kind to provide a fundamental insight into the mechanisms that link central nervous system hypoxia, increased sympathetic activity, and high systemic arterial blood pressure.

### What Is Relevant?

- Brainstem hypoxia contributes to the development of systemic arterial hypertension.
- Signaling pathways activated by central hypoxia represent a potential therapeutic target for the treatment of systemic arterial hypertension.

### Summary

Low PO_2_ of the brainstem parenchyma may be associated with higher levels of ambient ATP and lactate within the presympathetic circuits, leading to increased central sympathetic drive and concomitant sustained increases in the arterial blood pressure.
